# The Roles of Exosomes in Metastasis of Sarcoma: From Biomarkers to Therapeutic Targets

**DOI:** 10.3390/biom13030456

**Published:** 2023-03-01

**Authors:** Linyun Tan, Yitian Wang, Xin Hu, Li Min

**Affiliations:** Department of Orthopedics, Orthopedic Research Institute, West China Hospital, Sichuan University, 37 Guoxue Road, Chengdu 610041, China

**Keywords:** sarcoma, metastasis, exosome, diagnostic biomarker, therapeutic target

## Abstract

Sarcoma is a heterogeneous group of mesenchymal neoplasms with a high rate of lung metastasis. The cellular mechanisms responsible for sarcoma metastasis remain poorly understood. Furthermore, there are limited efficacious therapeutic strategies for treating metastatic sarcoma. Improved diagnostic and therapeutic modalities are of increasing importance for the treatment of sarcoma due to their high mortality in the advanced stages of the disease. Recent evidence demonstrates that the exosome, a type of extracellular vesicle released by virtually all cells in the body, is an important facilitator of intercellular communication between the cells and the surrounding environment. The exosome is gaining significant attention among the medical research community, but there is little knowledge about how the exosome affects sarcoma metastasis. In this review, we summarize the multifaceted roles of sarcoma-derived exosomes in promoting the process of metastasis via the formation of pre-metastatic niche (PMN), the regulation of immunity, angiogenesis, vascular permeability, and the migration of sarcoma cells. We also highlight the potential of exosomes as innovative diagnostic and prognostic biomarkers as well as therapeutic targets in sarcoma metastasis.

## 1. Introduction

Sarcoma is a group of large heterogeneous and primary malignant tumors, accounting for 19–21% of all cancer-related deaths in children and adolescents due to their highly aggressive nature and systemic metastasis [1,2]. Surgical excision, chemotherapy, immunotherapy, and targeted therapy are the conventional therapeutic strategies for patients with metastatic sarcoma [3,4,5]. However, the prognosis for patients with metastatic sarcoma has remained stagnant over the past three decades, with a 5-year survival rate of only 16% despite systemic therapy [6,7,8,9,10]. The validated diagnostic biomarkers and therapeutic targets still do not meet clinical needs for the treatment of metastatic sarcomas, including osteosarcoma, chondrosarcoma, Ewing sarcoma, liposarcoma, and rhabdomyosarcoma [11,12,13,14,15].

Exosomes belong to a category of extracellular vesicles which play critical roles in intercellular information transmission [16,17,18,19]. Constituted with various biomolecules, exosomes are involved in many physiological and pathological processes via multiple mechanisms, including transporting cargos, influencing signaling pathways, and changing cell behaviors [20,21,22,23]. Additionally, the relatively stable bioactivity and specificity of exosomes can prevent the contents enclosed within exosomes from degrading [24,25].

Exosomes are widely recognized as essential modulators of cancer metastasis, and are a source for identifying potential biomarkers and therapeutic targets for many malignant tumors [26,27,28]. Several reviews have focused on exosomes in metastatic neoplasms (including lung cancer, breast cancer melanoma, and prostate cancer), but none have discussed the role of these vesicles in metastatic sarcomas [29,30,31,32,33,34]. The detailed molecular mechanisms of exosomes in sarcoma metastasis still need further investigation.

In this review, we discover exosome-related mechanisms in sarcoma metastasis and discuss the potential of exosomes as diagnostic and prognostic biomarkers, as well as potential therapeutic targets for metastatic sarcomas in the future.

## 2. The Mechanisms of Exosome Involvement in Sarcoma Metastasis

Exosomes are extracellular vesicles excreted by mammalian cells through endosomes that are of vital importance in cell communication by transporting intercellular cargo [20]. Exosomes are characterized by a 30–150 nm size and cup-shaped appearance under electron microscopy [35]. They carry proteins, nucleic, acids lipids, and other substances which are widely distributed in a variety of body fluids, such as plasma, urine, chest and abdominal fluid, bile, saliva, and cerebrospinal fluid [36]. When they were first discovered by Johnstone et al. [37], they were considered to be related to the removal of cellular waste, but several studies have since illustrated that exosomes are essential to cellular communication between adjacent cells and at great distances [21]. Exosomes modulate the activity of sarcoma cells to distant metastases by delivering molecularly active noncoding RNAs (ncRNAs), including miRNA and long noncoding RNA (lncRNA) [38,39]. These RNA molecules, together with proteins, act as genetic materials and play vital roles in intercellular communication. In-depth studies show that two main molecular mechanisms are involved in exosomal-modified metastasis: the translational repression of anti-oncogenes by exosomal miRNAs and a lack of translational oncogene repression based on the sponging of miRNAs through lncRNAs [39].

**Figure 1:**
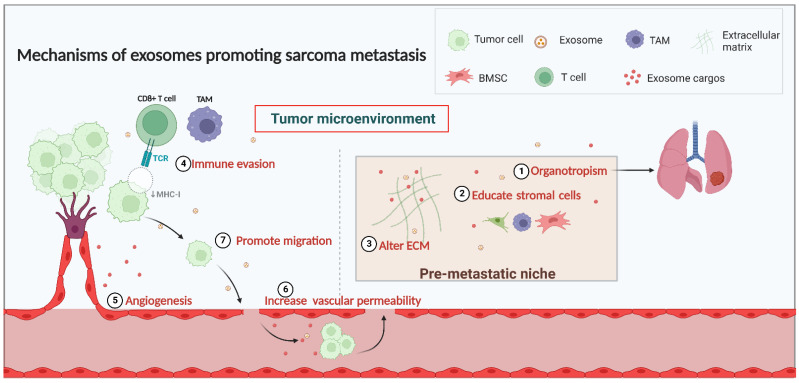
Mechanisms of exosomes in sarcoma metastasis.

The tumor microenvironment (TME) is critical in facilitating sarcoma growth and metastasis, and an increasing body of literature illustrates that the pre-metastatic niche (PMN) is intimately linked with the tumor cells metastasizing [40]. PMN is an environment in a target organ that can be conducive to the metastasis of a primary tumor [29,41]. Before metastasis, the second target organ can be specially modified by exosomes to establish favorable conditions for circulating sarcoma cells’ growth, known as PMN, and eventually establish metastasis [42]. The establishment of PMN involves organotropism, interaction with the stromal cells, and alterations in the extracellular matrix [42,43,44]. Moreover, exosomes can transport proteins or nucleic acids from parental to recipient cells, which induces immunomodulation, angiogenesis, and tumor cell migration, thereby facilitating sarcoma metastasis [45,46] (Figure 1).

### 2.1. Exosomes and the TME in Sarcoma Metastasis

The tumor microenvironment is a complex internal environment on which tumor cells arise and depend, mainly composed of the tumor itself and some other non-tumor cells (such as immune cells, fibroblasts, inflammatory cells, and other cells), as well as blood vessels, extracellular matrix, and biomolecules [47]. The TME generally plays a vital role in the processes of sarcoma growth, progression, and metastasis [48,49]. By cooperating closely with immune cells and stromal cells in the TME, tumor cells contribute to tumor immune escape and chronic inflammation regulation [45]. The exosome secreted by sarcoma cells is called the sarcoma-derived exosome. The information sharing between sarcoma and host cells is mainly achieved by the sarcoma-derived exosome [18]. The sarcoma-derived exosome contains various inhibitory molecules involved in negatively modulating the immune response, which can activate several cellular inhibitory pathways so that sarcoma cells can reprogram the immune cell functions [50]. Moreover, during tumor progression, sarcoma-derived exosomes have been proven to drive metastasis via angiogenesis, vascular permeability, and the migration of spreading sarcoma cells [48,50,51].

### 2.2. Exosome and PMN Formation in Sarcoma Metastasis

The PMN is a preformed microenvironment made by exosomes secreted by the primary tumor site before widespread metastasis [52,53]. Exosomes optimize the environment for sarcoma colonization, outgrowth, and metastasis [54]. Sarcoma metastasis is a multistep and complex biological process [55]. It is becoming clear that metastasis is not solely a consequence of autonomous sarcoma cell properties, but also a complex interplay between tumor cells and the many components of the metastatic microenvironment, including the extracellular matrix, inflammatory cells, and stimulatory molecules [56]. It is now well established that primary sarcoma cells release exosomes that promote the preparation at future metastatic sites prior to circulating sarcoma cell colonization and thereby favor the establishment of specialized microenvironments, designated as the PMN [42,57]. The formation of PMN in sarcoma is a prevalent precondition of metastasis [40,48].

#### 2.2.1. Organotropism in PMN Formation

Exosomes can induce metastatic sarcoma organotropism via the release and uptake of exosomal cargos [42]. Highly metastatic sarcoma can secret more organotropic exosomes than lowly metastatic sarcoma [58]. Moreover, sarcoma-cell-derived exosomal integrins (ITGs) govern organ-specific metastasis by fusing with target cells in a tissue-specific fashion, thereby initiating PMN formation [44,59,60]. ITGs consist of α and β subunits and are embedded in the membrane [59]. They interact with extracellular matrix (ECM) proteins and are involved in cell adhesion, transplanting exosomes into specific cells, such as lung fibroblasts and epithelial cells in the lung, thus encouraging lung metastasis [44]. For instance, the exosomal integrins ITG αvβ3 can selectively adhere to ECM-enriched cellular areas in the lung and induce the upregulation of genes associated with pulmonary metastasis and inflammation [61]. In particular, research on ITG expression in other tumors has also demonstrated that this protein can only facilitate sarcoma cell adhesion to pulmonary vessels and does not promote tumor cell proliferation [62,63].

#### 2.2.2. Interaction with Stromal Cells in PMN Formation

The survival of cancer cells that metastasize from primary tumors to secondary sites depends upon the stroma microenvironment. Tumor-derived exosomes assist in this process by educating and remodeling stromal cells in the metastatic site to support tumor cell viability and metastatic dissemination [64,65,66]. Functionally, they reprogram stromal cells in the PMN, including cancer-associated fibroblasts (CAFs), mesenchymal stem cells (MSCs), and tumor-associated macrophages (TAMs) [67,68,69].

MSCs interact with cancer and other cells in the TME via paracrine factors and through exosomes to support tumor growth, progression, and metastasis [70]. Previous work concerning MSCs in sarcoma was conducted in mainly osteosarcoma (OS), which are assumed to originate at some stage in the differentiation process of MSCs to pre-osteoblasts [71,72,73]. The osteogenic differentiation stage of bone marrow MSCs (BMSCs) inflicts the phenotype of in vivo sarcoma development, implying that BMSC-derived osteogenic progenitors might be the origin cells for OS [74]. Exosomes derived from BMSCs are reported to promote cell proliferation, migration, and the invasion of OS cells by promoting oncogenic autophagy [73]. Exosomes secreted by BMSCs also protect OS cells from apoptosis and drive tumorigenesis and metastasis [75]. However, the current research has mainly focused on the effect of BMSCs on sarcoma, with few related studies on other tissue-derived MSCs, such as adipose-derived MSCs (AD-MSCs), dental-pulp-derived MSCs (DP-MSCs), and human umbilical cord MSCs (HUC–MSCs).

CAFs are unique reprogrammed stromal cells with roles in pre-metastatic niche formation and metastasis, which is a major component of cancer stromal cells that account for about 40∼50% of the total cell population in cancer [76]. Tumor-cell-derived exosomes promote the activation of CAFs and encourage CAF recruitment. CAFs have been reported to contribute to tumor invasiveness and metastasis by secreting a tumorigenic cytokine milieu of TGF-β, stromal-cell-derived factor-1α (SDF-1α), S100A4, fibronectin, and matrix metalloproteinases in the local stromal cell microenvironment [77,78].

TAMs are of the anti-inflammatory (M2) subtype and permeate malignant tissues [79]. Within the tumor microenvironment, TAMs secrete IL4, IL-5, and IL-6, which cannot only facilitate invasion and metastasis, but also promote tumorigenesis, angiogenesis, matrix remodeling, and immune system suppression [80]. One recent study showed miR-221-3p is a miRNA that is highly expressed in metastatic OS [67]. When miR-221-3p is phagocytosed by undifferentiated macrophages, it undergoes M2 polarization by curbing the suppressor of cytokine signaling SOCS3 and activating the JAK2/ signal transducer and activator of the transcription 3 (STAT3) pathway [67,80]. These emerging works have supported the role of directing the M2 subtype at multiple stages of tumorigenesis and pre-metastatic niche formation.

#### 2.2.3. Alterations in the ECM in PMN Formation

Exosomes are important intercellular communicators, which do not only account for cells, but also for the ECM, which is an important constituent of non-transformed tissues as well as tumors [81]. Exosomes derived from the metastatic tumor can promote tumors to acquire the capability of metastasis via remodeling the ECM [82]. By binding to individual components of the ECM, exosomes, which are rich in proteases, modulate the ECM, as demonstrated for collagen IV, laminin332, and hyaluronic acid (HA) degradation [81,83]. These changes are promoted by exosomes to create a suppressive environment for metastatic tumor cell seeding and growth. The integration of OS-derived exosomes and OS cells has been proven to modulate the ECM and induce fibronectin deposition in PMN [84]. For instance, OS-derived exosomes exhibit a-SMAs, markers associated with high fibronectin and fibroblast, to enhance the expression of lncRNA SNHG17 in OS to encourage metastasis [69].

### 2.3. Regulation of Immunity

The immune system is a significant barrier to metastasis. For sarcomas to thrive in their new environment, it is of utmost importance for the PMN to protect the metastatic sarcoma cells from being apoptosed upon entering the metastatic site [85]. Exosomes produced by tumor cells can support their escape from immune surveillance [86,87,88]. Exosomes can upregulate the expression of proinflammatory factors and induce the secretion of some proinflammatory chemokines and cytokines, such as IL10, TGF-β2, and IL-35, in the tumor local inflammatory microenvironment, thus weakening antitumor immune responses [87,89,90]. These factors recruit TAMs, tumor-associated neutrophils (TANs), regulatory T cells (Tregs), and myeloid-derived suppressor cells (MDSCs) to distant secondary sites in cooperation with the tumor-derived exosome, thereby suppressing anti-tumor immune responses [91]. In more cases, exosomes interfere with immune recognition and inhibit the function of immune cells, leading to immune escape and sarcoma metastasis. For example, in exosomes of OS-bearing dogs, the expression of the plasma protease C1 inhibitor increased and the expression of C1qa decreased, which could prevent the activation of classical pathways and facilitate immune escape [92]. Recent studies have demonstrated that metastatic OS-cell-derived exosomes can induce the M2-type differentiation of macrophages largely through Tim-3 mediation and create an immunosuppressive tumor-promoting microenvironment through the production of cytokines, including IL-10, TGF-β, and VEGF [93].

### 2.4. Angiogenesis and Vascular Permeability

Angiogenesis is the formation of new blood vessels in capillaries or venules behind capillaries [94,95]. Tumor angiogenesis is characterized by the excess of pro-angiogenic factors that lead to uncoordinated endothelial cell (EC) proliferation and supportive cell migration [96]. This process is regulated by the interaction between proangiogenic and antiangiogenic factors. Although these factors are stable under normal physiological conditions, they can be activated or inactivated by external stimuli [97,98]. Sarcoma-derived exosomes are associated with an important mechanism that promotes angiogenesis. One such study demonstrated their ability to enhance the viability and migration of human umbilical vein endothelial cells. At the molecular level, proteomics has shown an increased expression of proangiogenic factors, including VEGF-A, IL-6, and IL-8 mRNAs, as well as proteins within OS exosomes to upregulate angiogenesis [46]. Chondrosarcoma (CS)-cell-derived exosomes carrying lncRNA RAMP2-AS1 are present within the serum of chondrosarcoma patients and are robust activators of angiogenesis. Mechanistically, chondrosarcoma-derived exosomes transport RAMP2-AS1 into human umbilical vein endothelial cells (HUVECs), prompting a signaling cascade that binds miR-2355-5p and promotes the expression of VEGFR2; this stimulates endothelial cell growth [46]. In addition to participating in angiogenesis, exosomes can encourage tumor metastasis by increasing vascular permeability [48,99]. Vascular endothelial cells maintain vascular barrier function through adhesion and tight connections and provide a physical barrier for substances inside and outside the vessel [100]. Exosomes can damage the connection between epithelial cells and alter blood vessel permeability to enhance sarcoma-distant metastasis [44,101]. In breast cancer, exosomal miR-105 in metastatic tumor cells induces the destruction of the vascular endothelial barrier in the metastasis pathway, increasing vascular permeability and promoting distant metastasis [102]. The regulation of vascular permeability by sarcoma-derived exosomes is not widely described in the literature. However, we can make a reasonable speculation that they are closely related.

### 2.5. Migration of Sarcoma Cells

Exosomes derived from sarcoma cells or sarcoma-educated stromal cells play significant roles in metastasis by promoting the migration ability of sarcoma cells [103]. JNK/p38-MAPK, PVT1/ERG, programmed cell death 4 (PDCD4)/ERK1/2, SOCS3/JAK2/STAT3, TGF-β/COL6A1, IL6/STAT3, PI3K/Akt/mTOR, and Hedgehog in exosomes regulate genes or signaling pathways to promote the migration and metastasis of sarcoma cells [48]. For instance, Hedgehog overexpression is associated with the aggressive behavior of OS. Exosomes derived from hBMSCs activate Hedgehog signaling in human osteosarcoma MG63 cells and stimulate the migration and proliferation of OS cells [104]. Additionally, specific sarcoma-associated exosomal miRNAs, including miR-769-5p, miR-1307, miR-486-5p, miR-208a, miR-221-3p, miR-1228, miR-21-5p, and miR-675, act as important regulators by playing an enabling role in promoting the migration of sarcoma cells [48]. For instance, exosome-derived miRNA-221-3p can aggravate the malignant behaviors of OS by targeting SOCS3 and can promote the proliferation and migration of OS cells by regulating the JAK2/STAT3 signaling pathway [67].

## 3. Exosomes as Diagnostic and Prognostic Biomarkers for Sarcoma Metastasis

**Figure 2:**
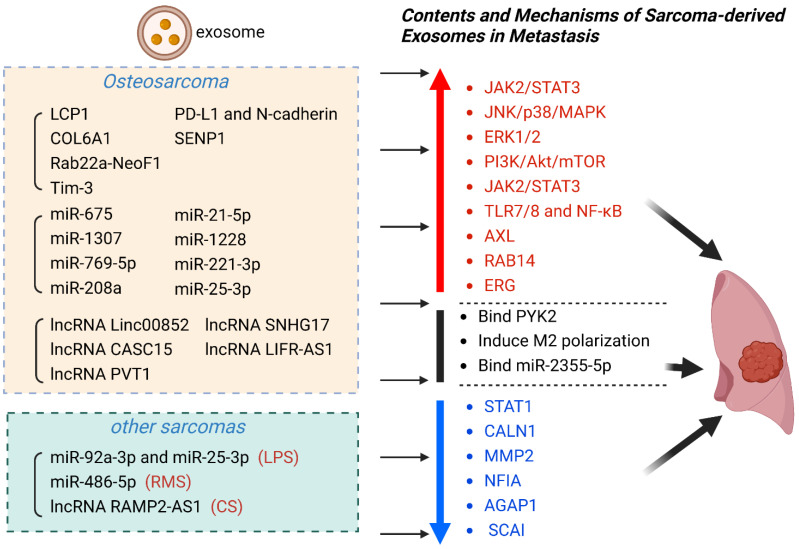
Sarcoma-derived exosome contents and mechanisms for metastasis. Exosomes are secreted vesicles that are encapsulated by a lipid bilayer and contain various biomolecules such as protein (LCP1, COL6A1, etc.), miRNA (miR-21-5p, miR-1228, etc.), as well as lncRNA (Linc00852, SNHG17, etc.). To enable metastasis, exosomes upregulated and downregulated related pathways. Up- and downregulated pathways are highlighted in red and blue, respectively.

Exosomes play an integral part in sarcoma metastasis, participating in sarcoma cell proliferation, invasion, and migration. With the in-depth research in this emerging field, the potential clinical value of exosomes in sarcoma metastasis has been gradually excavated. The surface markers on sarcoma-derived exosomes and internal components mirror many of the molecular features of sarcoma which it originates from. Meanwhile, the unique expression pattern and relatively stable content make exosomes promising candidates as novel biomarkers for sarcoma metastasis [19,29,105]. Therefore, exosomes play potential roles in early diagnosis and prognosis prediction in sarcoma metastasis (Table 1) (Figure 2).

### 3.1. Osteosarcoma

OS is the most common primary tumor of long bones affecting children and adolescents [121]. Due to the high heterogeneity and complex nature of the tumorigenesis and development of OS, early diagnosis and surveillance for metastasis are urgently needed. Growing evidence implicates that exosomes can contribute to metastasis in OS, and the diversity of exosome cargos can be considered as a biomarker of OS metastasis.

#### 3.1.1. Exosomal Proteins as Biomarkers in OS

LCP1, an actin-binding protein, was found in serum exosomes from OS patients and the exosomal LCP1 level was correlated with pulmonary metastasis [106]. Exosomal LCP1 was transferred by OS-derived exosomes to BMSCs, resulting in neuregulin receptor degradation protein-1 (Nrdp1) becoming destabilized and subsequently activating the JAK2/STAT3 signaling pathway to promote lung metastasis [106]. A recent study has demonstrated that collagen type VI alpha 1 (COL6A1) expression is significantly upregulated in OS tissues when compared to non-tumor tissues, especially in lung metastasis tissues [65]. Moreover, the upregulation of COL6A1 was significantly related with poor prognosis in patients with OS [65]. Mechanistic analysis has showed that alterations in COL6A1 promoted the ubiquitination and proteasome degradation of signal transducers and activators of transcription 1 (STAT1) to stimulate OS lung metastasis [65]. Additionally, exosomal COL6A1 derived from OS cells transform normal fibroblasts into CAFs by secreting pro-inflammatory cytokines, including IL-6 and IL-8, and the CAFs can then promote OS metastasis by mediating the TGF-β/COL6A1 signaling pathway [65].

Another study found that a new fusion protein, Rab22a-NeoF1, can be sorted into exosomes of OS cells to facilitate the lung PMN formation and consequently promote lung metastasis [122]. To be more specific, Rab22a-NeoF1 in the exosome promotes OS cell metastasis through the activation of Ras homolog gene family member A (RhoA) by its binding partner PYK2 [122]. In addition, the exosomal PYK2 increases the activation of STAT3/RhoA to induce TAMs into the M2 phenotype [122]. Furthermore, Tim-3 was a type of T cell immunoglobulin and mucin domain (Tim) family protein [93]. Upon uptake by OS cells, exosomal Tim-3 can induce the M2 polarization of TAMs and activate the intracellular expression of TGF-β, IL-10, and VEGF, leading to the lung metastasis of sarcoma by promoting migration, invasion, and EMT [93].

#### 3.1.2. Exosomal miRNAs as Biomarkers in OS

Micro-RNAs (miRNAs) are single-chain, endogenous, and small noncoding RNAs with a length of approximately 22–24 nucleotides [123]. They regulate gene expression at the transcriptional and post-transcriptional level by binding the seed sequence to the target messenger RNA (mRNA), and then repress protein translation or promote mRNA degradation to contribute to the spontaneous metastasis of OS [124]. Exosomal miRNAs have emerged as promising biomarkers with potential applications in the diagnosis and prognosis of invasion and metastasis of OS.

MiR-675 is overexpressed in cancer tissues from metastatic OS patients as well as metastatic OS cell lines [103]. OS-derived exosomes containing miR-675 can induce proliferation and invasion, increasing the migration of non-malignant fibroblast cells by decreasing the expression of calneuron 1 (CALN1), which results in sarcoma growth and metastasis [103]. Similarly, miR-1307 is overexpressed in highly invasive OS cells, where it is assimilated into the exosome [110]. Upon uptake by OS cells, exosomal miR-1307 then promotes the proliferation, migration, and invasion of OS cells by inhibiting the expression of ankyrin repeat and PH domain 1 (AGAP1) [110].

A growing number of studies suggest that miRNAs can be delivered to OS cells by BMSC-derived exosomes during OS metastasis. Exosomes produced by BMSCs could stimulate sarcoma cells to express chemokines, in turn promoting the proliferation and migration of OS cells. For instance, BMSC-derived exosomes containing miR-769-5p could be taken up by OS cells and induce the EMT and lung metastasis of OS xenografts in nude mice by increasing E-cadherin expression, suppressing dual-specific phosphatase 16 (DUSP16), and activating the JNK/p38 MAPK signaling pathway [111]. In addition, BMSCs in the metastatic OS patient present high levels of miR-208a [112]. Serum miR-208a-positive exosomes can induce invasive growth of OS cells by counteracting with PDCD4 to activate the ERK1/2 signaling pathway, thus promoting the malignant migration of OS cells [112]. Therefore, exosomal miR-208a may be considered a putative marker to predict OS metastatic progression [112]. Another study demonstrated that BMSCs could produce miR-21-5p-positive exosomes to induce the cell proliferation and migration of OS cells by downregulating the expression of PIK3R1 mRNA, decreasing p85α protein production, and activating corresponding PI3K/Akt/mTOR signaling cascades [113].

In addition to BMSCs, CAF-derived exosomal miR-1228, internalized by OS, could downregulate endogenous SCAI mRNA and protein levels in OS, thus promoting the proliferation and metastasis of OS [64,125]. TAMs in OS are largely M2 phenotype macrophages, and M2-type TAMs have been associated with OS metastasis and poor prognosis [64,126]. It has been verified that M2 macrophage-derived miR-221-3p inhibited SOCS3 gene expression and activated the JAK2/STAT3 signaling pathway, therefore aggravating the proliferation and metastasis of OS [67].

#### 3.1.3. Exosomal lncRNAs as Biomarkers in OS

Besides the distinct miRNAs previously mentioned, cancer tissues or peripheral blood originating from metastatic OS patients have presented high levels of long noncoding RNAs (lncRNAs). LncRNAs constitute a novel class of non-coding transcript RNAs with unique characteristics [127]. These RNAs are implicated in OS survival, proliferation, invasion, and metastasis [128].

Exosomal lncRNA linc00852 was found in cell culture fluid from OS cells, and exosomal lncRNA linc00852 could promote OS metastasis by increasing the expression of receptor tyrosine kinase AXL [117]. Mechanistic analysis has revealed that exosomal lncRNA linc00852 may contribute to OS invasion and migration by inducing the invasive growth of OS cells as a competitive endogenous RNA (ceRNA) for miR-7-5p and upregulating AXL expression in OS cell cytoplasm [117]. Similarly, metastatic OS patients presented high plasma long noncoding RNA (lncRNA) cancer susceptibility candidate 15 (CASC15) levels [118]. Serum exosomal lncRNA CASC15 could induce the invasive growth and metastasis of OS cells by counteracting with miR-338-3p to upregulate RAB14 expression [118].

By acting as a miR-183-5p sponge and activating ERG expression, exosomal lncRNA PVT1 secreted from BMSCs stimulates the proliferation and metastasis of OS cells [119]. The lncRNA SNHG17 was also highly abundant in exosomes derived from CAFs, and it was transported into OS cells [69]. As a ceRNA, lncRNA SNHG17 could inhibit the expression of matrix metallopeptidase 2 (MMP2) by binding with miR-2861 to promote OS progression and metastasis. [69]. In addition, it has been demonstrated that exosomal lncRNA LIFR-AS1 could be directly transferred from macrophages to OS cells to promote cell migration and metastasis [120]. The lncRNA LIFR-AS1 derived from M2 TAM enhanced EMT and inhibited apoptosis in OS cells [120]. Mechanistic analyses have demonstrated that exosomal lncRNA LIFR-AS1 promoted OS metastasis and proliferation via sponging miR-29a and downregulating NFIA expression [120].

### 3.2. Chondrosarcoma

Chondrosarcoma (CS) is the second most common primary bone tumor among all malignant bone tumors. Chondrosarcoma is a malignant tumor of cartilage origin that includes several subtypes depending on their histological characteristics and pathological sites. Unlike many tumors, chondrosarcoma is usually resistant to chemotherapy and radiotherapy. Exosomes have shown their ability to regulate human chondrosarcoma metastasis.

In chondrosarcoma tissues and cell lines, lncRNA RAMP2-AS1 expression was upregulated [46]. Elevated expressions of RAMP2-AS1 are implicated in the poor prognosis of CS and angiogenesis of HUVECs [46]. In vitro experiments showed that the knockdown of RAMP2-AS1 efficiently decreased the recipient of HUVECs [46]. Mechanistically speaking, exosomes derived from CS transport RAMP2-AS1 into HUVECs, competitively bind miR-2355-5p, and promote the expression of VEGFR2, resulting in angiogenesis [46]. Additionally, elevated serum RAMP2-AS1 levels were correlated with advanced and metastatic characteristics of chondrosarcoma patients, indicating that RAMP2-AS1 appears in exosomes as a novel therapeutic target of chondrosarcoma.

### 3.3. Rhabdomyosarcoma 

Rhabdomyosarcomas (RMSs) are a heterogeneous group of malignant tumors and are the most frequent soft tissue sarcoma in children [129]. There are two major subtypes—embryonal RMSs (ERMSs) and alveolar RMSs (ARMSs). ERMS typically occurs in children under 10 years old and shows a histology similar to embryonic skeletal muscle.

MiR-486-5p, an effector of PAX3-FOXO1, promotes migration, invasion, and colony formation of rhabdomyosarcoma cells by regulating its paracrine effects in exosomes. [116]. Human RMS cell analysis showed numerous enriched miR-486-5p in both cells and exosomes and, to a higher extent, in ARMS subtypes [116]. The analyses of the human serum sample showed that miR-486-5p is enriched in the RMS-patient-derived exosomes of patients with RMS, and follow-up after chemotherapy showed a decrease in control values [116]. So, there is a novel role of both PAX3-FOXO1 and its downstream effector miR-486-5p in exosome-mediated oncogenic paracrine effects of RMS, making it an attractive biomarker in the metastasis and prognosis of rhabdomyosarcoma [116].

### 3.4. Liposarcoma

Liposarcoma (LPS) is the most common soft tissue sarcoma, accounting for roughly 25% of all sarcomas in adults [130]. Despite surgery and certain drugs (such as doxorubicin, ifosfamide, antimitotic docetaxel, and antimetabolite gemcitabine) seeming helpful, metastatic liposarcoma patients have a poor prognosis. Despite the development of combined modality treatments against LPS in recent years, a significant proportion of patients respond only modestly to such approaches, possibly contributing to local or distant recurrence. The early detection of recurrent or metastatic disease could improve patient prognosis by triggering earlier clinical intervention. MiR-25–3p and miR-92a-3p are secreted by LPS cells through exosomes and may be useful as potential biomarkers of LPS metastasis [115]. Mechanistically, miR-25–3p and miR-92a-3p stimulated the secretion of pro-inflammatory cytokine IL-6 from tumor-associated TAM in a TLR7/8-dependent manner, in turn promoting LPS cell proliferation, invasion, and metastasis via this interaction with the surrounding microenvironment [115].

## 4. Therapeutic Strategies Based on Exosome for Sarcoma Metastasis

**Figure 3:**
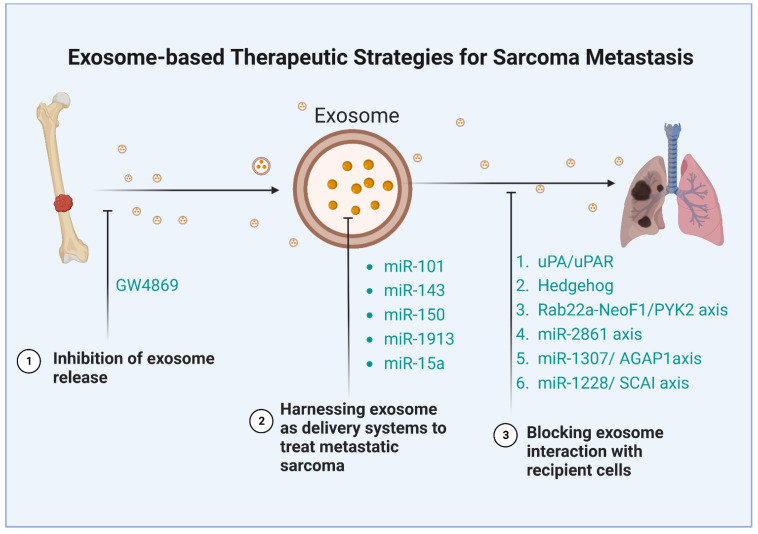
Therapeutic strategies using exosomes in sarcoma metastasis.

The critical functions of exosomes and their cargos in metastasis demonstrate their potential as therapeutic targets, although this field of research is still in its early stages. It has already been shown that exosomes can be utilized in medicine as therapeutic tools, and their lipid bilayer makes them perfect drug transport systems [131]. Sarcoma-derived exosomes can activate critical signaling pathways in the recipient cells and interact with local and distant cells to promote sarcoma invasion and metastasis. Consequently, targeting these exosomes as a therapeutic approach in metastatic sarcoma treatment seems reasonable. There have been several strategies developed to counter the effects of these exosomes: inhibiting the secretion of exosome, blocking the downstream signaling pathways of the exosome, and harnessing exosomes as carriers to treat metastasis (Table 2) (Figure 3).

### 4.1. Inhibition of Exosome Release

Given the fact that sarcoma cells send out exosomes to encourage their metastasis and proliferation, the first idea is to inhibit the release of metastatic sarcoma exosomes. Recent studies have identified that the neutral sphingomyelinase inhibitor GW4869 functionalized by inhibiting the ceramide-mediated inward budding of multivesicular bodies (MVBs) to decrease exosome release [139,140]. So, GW4869 treatment can significantly decrease the amount of OS-cell-secreted exosomes [69]. Another study indicated that metastatic OS cells lines SJSA-1 and HOS with GW4869 treatment significantly inhibited OS cell viability and migration when compared with the control group [69]. In another study, GW4869 effectively decreased exosome release in OS cells and led to an increase in E-cadherin and a decrease in vimentin and N-cadherin expression, thus delaying tumor growth and lung metastasis [111].

Similar results can be obtained according to the same experimental process in LPS [115]. Through adding GW4869 in LPS cell culture media, researchers found that the content of exosome-secreted miR-25-3p and miR-92a-3p was strongly impaired and the exosome-mediated lung metastasis activity was diminished [115]. However, these studies did not provide any in vivo antitumor efficacy assessment. More in vivo and clinical trials that can observe metastasis inhibition may be worthy of further investigation.

### 4.2. Blocking Downstream Pathway of Exosome

An alternative therapeutic approach aimed at inhibiting exosome function is by blocking their downstream signaling pathways. IL-6/STAT3 is an essential pathway involved in the progression of metastatic sarcoma, and IL-6 production via sarcoma exosomes made mesenchymal stem cells responsible for stimulating sarcoma metastasis [141]. So, blocking IL-6/STAT3 may be an excellent approach used to suppress sarcoma metastasis [141]. For instance, tocilizumab, a therapeutic monoclonal antibody used against IL-6R, inhibited OS invasion and metastasis by downregulating IL6 [132]. Moreover, another recent study concluded that inhibition of the urokinase plasminogen activator (uPA) and the uPA receptor (uPAR) axis would be a new therapeutic strategy [133]. When metastatic OS cells were upregulated, uPA and uPAR were co-cultured with non-metastatic OS cells with a low expression of uPA and uPAR because uPA was transmitted between two cells lines using the exosomes, and the level of uPA and uPAR in non-metastatic OS cells was significantly increased, thereby effectively enhancing the proliferation rate and metastasis of non-metastatic OS cells [133]. WX-340 is a small-molecule competitive active-site uPA enzymatic inhibitor [142]. The WX-340 treatment of KHOS tumor-bearing mice significantly reduced pulmonary metastasis in the experimental animals compared to vehicle controls when administered early. Importantly, a similar result was obtained in in vivo experiments [133]. Using xenograft OS models, researchers found that the injection of small-molecule inhibitors of uPA into primary OS mice significantly reduced pulmonary metastasis in the experimental animals compared to vehicle controls [132,142]. Another study also highlighted that inhibiting Hedgehog signaling could be considered as a therapeutic intervention to reduce OS progression [104].

As discussed earlier, the lung metastases of OS cells were promoted by the exosomal Rab22a-NeoF1 fusion protein, and this event could be targeted by disrupting its interaction with PYK2 using a designed internalizing RGD peptide [107]. The overexpression of exosomal miR-2861 has also been demonstrated to reverse the activation of SNHG17 on MMP2 expression [69]. Inhibiting the miR-2861 axis resulted in preventing OS proliferation and metastasis [69]. In addition, a recent study noted that during the metastasis of OS, the binding of miR-1307 to the 3′ untranslated region of AGAP1 played an essential role, and this made AGAP1 targeting a new therapeutic target for OS patients and provided new therapeutic ideas [110]. Exosomal miR-1228 controlled OS cell migration and invasion through SCAI [64]. The increased expression of SCAI could inhibit OS cells’ metastasis and migration [64].

Altogether, the mechanisms of blocking the downstream pathways of exosomes are regarded as significant therapeutic methods in resisting metastatic sarcoma. Moreover, the results might help to find new therapeutic targets and novel opportunities for preventing sarcoma metastasis.

### 4.3. Harnessing Exosomes as Delivery Systems to Treat Sarcoma Metastasis

Exosomes have the potential to be excellent carriers in delivering anti-cancer biomolecules for metastatic sarcoma because of their unique properties, such as high biocompatibility, low toxicity, specificity, and stability [143,144,145]. Experimental evidence showed that several exosomes from other sources can significantly reduce the progression and aggressiveness of sarcoma cells [143,145]. MSCs play a pivotal role. One study found that AD-MSC-derived exosomes could be used as vehicles to transfer miR-101 into OS cells, effectively reducing OS cell migration and metastasis in vivo [134]. In addition, the transfection of miR-143 into MSCs could produce exosomal miR-143 and transfer into OS cells, suppressing OS cell migration and metastasis [135]. In another experiment, miR-150 loaded in MSC-derived exosomes inhibited OS cell metastasis and induced cell apoptosis through targeting IGF2BP1 [136]. HBMSC-derived miR-1913 inhibited OS progression and metastasis in vivo by targeting NRSN2, which might serve as an alternative for the treatment of OS metastasis [137].

Similarly, exosomal miR-195 was formed by normal human chondrocytes and delivered to OS cells [146]. MiR-195 inhibited kinesin superfamily protein 4A (KIF4A) expression by directly targeting its 3′-untranslated region, thereby suppressing the metastasis and proliferation of OS cells [146]. Another study revealed that miR-15a was identified as a major cargo of serum-derived exosomes and could be internalized by OS cells [138]. Exosomal miR-15a inhibited the proliferation and invasiveness of OS cells in vitro by activating the p53 signaling pathway and suppressing the GATA2/MDM2 axis [138].

The studies mentioned above show that exosomes are potential candidates in the treatment of sarcoma metastasis.

## 5. Clinical Experience of Exosomes in Sarcoma Metastasis

The advantages of using exosomes as non-invasive methods for prognostic stratification and precise therapy are evaluated clinically. Since many promising results have been achieved in in vitro and animal models, the use of exosomes is considered to be one of the most hopeful approaches for metastatic sarcoma diagnosis and therapy. Notably, some clinical trials have already made important achievements. As shown by the Musculoskeletal Tumor Center of Peking University People’s Hospital, compared with healthy subjects or individuals with a 5-year progression of free OS, OS patients with lung metastases usually exhibited elevated exosomal programmed death ligand 1 (PD-L1) and exosomal N-cadherin levels in the serum [108]. Further evidence has demonstrated that the detection of exosomal PD-L1 and N-cadherin from sera of OS patients may predict lung metastasis progression for OS patients [108]. Similarly, in another clinic trial in China with 146 OS patients, exosome-derived sentrin SUMO-specific protease 1 (SENP1) levels in peripheral blood samples were increased in 88.33% of OS patients [109]. A higher SENP1 level was associated with poorer disease-free survival and overall survival [109]. A recent clinical study in Japan proved that circulating miR-25-3p levels were identified with an aberrantly higher expression in OS-cell-derived exosomes compared to normal-cell-derived exosomes [114]. Furthermore, serum miR-25-3p levels at diagnosis were correlated with patient prognosis and reflected tumor burden [114]. As mentioned above, according to the NanoString profiling on RNA samples isolated from peripheral blood plasma vesicles (PBVs) in 16 human LPS patient samples and 8 healthy controls, miR-25-3p and miR-92a-3p could serve as novel potential biomarkers for the prediction of metastasis in adult LPS patients [115].

These observations indicate that peripheral blood N-cadherin, PD-L1, SENP1, miR-25-3p, and miR-92a-3p levels may be used as clinical biomarkers for diagnosing sarcoma micrometastases and evaluating prognosis.

## 6. Conclusions and Future Perspectives

The current rapid expansion of molecular biological techniques on exosomes evidently demonstrates that exosomes play essential roles in sarcoma metastasis. This review described the possible mechanisms of exosomes in sarcoma metastasis, including organotropism, intercellular transmission, ECM alterations, immunosuppression, angiogenesis, and cell migration. Secondly, several sarcoma-derived exosomal biomarkers, including miRNAs, proteins, and lncRNAs, were reported with a desirable diagnostic value and the potential of predicting prognosis for sarcoma metastasis. Finally, it was elucidated that exosomes can be attractive targets for the development of metastatic sarcoma therapeutic methods. 

Although positive results have been achieved toward investigating the relationship of exosomes and sarcoma metastasis, the clinical application of exosomes in sarcoma metastasis does not go deep enough. Future studies on exosomes and sarcoma metastasis may focus on the following aspects: (1) probing more detailed mechanisms of exosomes in sarcoma metastasis; (2) discovering additional exosomal biomarkers in sarcoma metastasis; (3) strengthening research on other metastatic sarcomas, such as liposarcoma and Ewing’s sarcoma; (4) establishing effective methods for the isolation, quantification, and analysis of exosomes in the body fluids of patients; (5) exploring new exosome-based therapeutic approaches for metastatic sarcoma treatment; and (6) accelerating the translation of basic science discoveries to clinical trials. The use of exosomes as biomarkers and therapeutic targets of sarcoma metastasis is already being explored, and more positive breakthroughs will be promising.

## Figures and Tables

**Table 1:** Exosomal markers in sarcoma metastasis.

Exosomal Contents	Sarcoma	Exosome Source	Exosomal Cargo	Effect	Clinical Significance	Ref
proteins	OS	Serum	LCP1	Activate JAK2/STAT3	promote metastasis	[106]
	OS	Serum	COL6A1	Downregulate STAT1	promote metastasis	[65]
	OS	Cell culture fluid	Rab22a-NeoF1	Bind PYK2	promote metastasis	[107]
	OS	Cell culture fluid	Tim-3	Induce macrophage polarization	promote metastasis	[93]
	OS	Serum	PD-L1 and N-cadherin	Not mentioned	promote metastasis	[108]
	OS	Plasma	SENP1	Not mentioned	promote metastasis	[109]
miRNA	OS	Serum	miR-675	Downregulate CALN1	promote metastasis	[103]
	OS	Serum	miR-1307	Inhibit AGAP1	promote metastasis	[110]
	OS	Cell culture fluid	miR-769-5p	Activate JNK/p38/MAPK	promote metastasis	[111]
	OS	Cell culture fluid	miR-208a	Activate ERK1/2	promote metastasis	[112]
	OS	Cell culture fluid	miR-21-5p	Activate PI3K/Akt/mTOR;Express VEGF	promote metastasis	[113]
	OS	Cell culture fluid	miR-1228	Inhibit SCAI	promote metastasis	[64]
	OS	Cell culture fluid	miR-221-3p	Activate JAK2/STAT3	promote metastasis	[67]
	OS	Serum	miR-25-3p	Not mentioned	promote metastasis	[114]
	LPS	Plasma	miR-92a-3p and miR-25-3p	Activate TLR7/8 and NF-κB	promote metastasis	[115]
	RMS	Serum	miR-486-5p	Mediate PAX3-FOXO1	promote metastasis	[116]
lncRNA	CS	Serum	lncRNA RAMP2-AS1	Competitively bind miR-2355-5p	promote metastasis	[46,115]
	OS	Cell culture fluid	lncRNA Linc00852	Upregulate AXL	promote metastasis	[117]
	OS	Slasma	lncRNA CASC15	Upregulate RAB14	promote metastasis	[118]
	OS	Cell culture fluid	lncRNA PVT1	Upregulate ERG	promote metastasis	[119]
	OS	Cell culture fluid	lncRNA SNHG17	Downregulate MMP2	promote metastasis	[69]
	OS	Cell culture fluid	lncRNA LIFR-AS1	Downregulate NFIA	promote metastasis	[120]

**Table 2:** Exosome-associated therapeutic strategies for sarcoma metastasis.

Strategy	Sarcoma	Experiment	Mechanism	Ref
Inhibiting exosome release	OS	In vitro	Inhibit LncRNA SNHG17	[69]
	OS	In vitro	Inhibit miR-769-5p	[111]
	LPS	In vitro	Inhibit miR-25-3p and miR-92a-3p	[115]
Blocking exosome interactions with recipient cells	OS	In vivo	Block IL-6 or TGF-β	[132]
	OS	In vivo and in vitro	Block the uPA/uPAR axis	[133]
	OS	In vitro	Block Hedgehog signaling	[104]
	OS	In vivo and in vitro	Block the Rab22a-NeoF1/PYK2 axis	[107]
	OS	In vitro	Block the miR-2861 axis	[69]
	OS	In vitro	Block the miR-1307/AGAP1axis	[110]
	OS	In vitro	Block the miR-1228/SCAI axis	[64]
Harnessing exosomes as delivery systems to treat metastatic sarcoma	OS	In vivo and in vitro	miR-101	[134]
	OS	In vitro	miR-143	[135]
	OS	In vivo and in vitro	miR-150	[136]
	OS	In vitro	miR-1913	[137]
	OS	In vitro	miR-15a	[138]

## Abbreviations

Adipose-derived MSCs (AD-MSCs), ankyrin repeat and PH domain 1 (AGAP1), bone marrow MSCs (BMSCs), cancer-associated fibroblasts (CAFs), cancer susceptibility candidate 15 (CASC15), chondrosarcoma (CS), collagen type VI alpha 1 (COL6A1), calneuron 1 (CALN1), competitive endogenous RNA (ceRNA), dental-pulp-derived MSCs (DP-MSCs), dual-specific phosphatase 16 (DUSP16), extracellular matrix (ECM), human umbilical cord MSCs (HUC–MSCs), human umbilical vein endothelial cells (HUVECs), integrins (ITGs), kinesin superfamily protein 4A (KIF4A), liposarcoma (LPS), lymphocyte cytosolic protein 1 (LCP1), long noncoding RNAs (lncRNAs), messenger RNA (mRNA), mesenchymal stem cells (MSCs), myeloid-derived suppressor cells (MDSCs), microRNAs (miRNAs), matrix metallopeptidase 2 (MMP2), nuclear factor I/A (NFIA), neuregulin receptor degradation protein 1 (Nrdp1), osteosarcoma (OS), pre-metastatic niche (PMN), proline-rich kinase-2 (PYK2), programmed death ligand 1 (PD-L1), programmed cell death 4 (PDCD4), peripheral blood plasma vesicles (PBVs), rhabdomyosarcoma (RMS), ras homolog gene family member A (RhoA), small ubiquitin-related modifier (SUMO), sentrin SUMO-specific protease 1 (SENP1), suppressor of cancer cell invasion (SCAI), signal transducers and activators of transcription 1 (STAT1), signal transducer and activator of transcription 3 (STAT3), tumor microenvironment (TME), tumor-associated macrophages (TAMs), tumor-associated neutrophils (TANs), regulatory T cells (Tregs), T lymphocytes 3 (Tim-3), urokinase plasminogen activator (uPA), uPA receptor (uPAR).
